# A revised compartmental model for biokinetics and dosimetry of 2-[^18^F]FDG

**DOI:** 10.1186/s40658-023-00528-9

**Published:** 2023-02-08

**Authors:** Alexandra Kamp, Martin Andersson, Sigrid Leide-Svegborn, Dietmar Noβke, Sören Mattsson, Augusto Giussani

**Affiliations:** 1 Department of Medical and Occupational Radiation Protection, Federal Office for Radiation Protection (BfS), Ingolstaedter Landstrasse 1, 85764 Neuherberg, Germany; 2 Department of Radiation Physics, Institute of Clinical Sciences, Sahlgrenska Cancer Center, Sahlgrenska Academy, University of Gothenburg, Gothenburg, Sweden; 3 Medical Radiation Physics Malmö, Department of Translational Medicine, Lund University, Malmö, Sweden; 4 Department of Medical and Occupational Radiation Protection, Federal Office for Radiation Protection (BfS), Neuherberg, Germany

**Keywords:** Nuclear medicine, Fluorodeoxyglucose, Biodistribution, Absorbed organ dose, Effective dose

## Abstract

**Background:**

The aim was to review available biokinetic data, collect own experimental data, and propose an updated compartmental model for 2-[^18^F]FDG in the frame of the revision of the ICRP report on dose coefficients for radiopharmaceuticals used in diagnostic nuclear medicine.

**Methods:**

The compartmental model was developed based on published biokinetic data for 2-[^18^F]FDG. Additional data on urinary excretion in 23 patients (11 males, 12 females) undergoing whole-body PET/CT examinations were obtained within this study. The unknown biokinetic model parameters were derived using the software SAAM II and verified with a modified version of IDAC-Iodide. Dose coefficients for reference adults were calculated with the programme IDAC-Dose 2.1. A dynamic bladder model was employed for urinary bladder dosimetry.

**Results:**

The proposed model consists of following compartments: blood, heart wall, brain, liver, lungs, pancreas, spleen, kidneys, urinary bladder content and a generic pool compartment “Other”. The latter was introduced to account for 2-[^18^F]FDG in body organ and tissues besides the explicitly modelled ones. The model predictions showed a good agreement with experimental data. Urinary bladder wall received the highest absorbed dose coefficient of 7.5E−02 mGy/MBq under the assumption of initial urine volume of 100 ml, first voiding at 45 min p.i. and 3.75 h voiding intervals thereafter. The effective dose coefficient calculated according to the current dosimetry framework of ICRP amounted to 1.7E−02 mSv/MBq, compared to 1.9E−02 mSv/MBq in ICRP Publication 128.

**Conclusion:**

A compartmental model for 2-[^18^F]FDG was proposed and will be used to replace the descriptive biokinetic model of ICRP Publication 128. The revised model and the provided dose coefficients are expected to improve reference dosimetry for patients administered with 2-[^18^F]FDG.

**Supplementary Information:**

The online version contains supplementary material available at 10.1186/s40658-023-00528-9.

## Introduction

Radiation doses received by patients from medical procedures are assessed and reported for radiological protection purposes. In internal dosimetry, the estimates rely on computational methods only, since it is not possible to measure an absorbed dose from internal exposure within the body. The estimation of absorbed doses for organs and tissues thus requires information on the biokinetics of an administered radiopharmaceutical and on the anatomy of the patient’s body. The detailed mathematical formalism of such calculations is described, e.g. in [1, 2]. Dosimetry in diagnostic nuclear medicine is usually done at an organ or tissue level, applying available sets of specific absorbed fractions (SAFs) calculated under the assumption of a homogeneous activity distribution in an entire source region. The corresponding absorbed dose is computed likewise for an entire organ or tissue as target region. While in nuclear medicine therapy individual dosimetry is recommended, the dosimetry for patients administered with diagnostic radiopharmaceuticals is often based on the published absorbed and effective dose coefficients for reference individuals estimated assuming standard biokinetic behaviour. Thus, it is important to assure that the existing reference models and the dose coefficients are consistent with the up-to-date computation methodologies and the experimental data that become available over time, to perform the quality control of the models and revise them if necessary.

Fluorodeoxyglucose, or 2-deoxy-2-[^18^F]-fluoro-D-glucose (2-[^18^F]FDG), is the most commonly used diagnostic radiopharmaceutical for positron emission tomography (PET) imaging. It is a glucose analogue used in the characterisation of glucose metabolism for diagnosis or follow-up of cancer diseases, and for investigation of myocardial and cerebral glucose metabolism. Biokinetic models for 2-[^18^F]FDG are published together with the corresponding absorbed and effective dose coefficients in the Publication 128 of the International Commission on Radiological Protection (ICRP) [2] and in the dose estimate report No. 19 by the Committee on Medical Internal Radiation Dose (MIRD) [3].

The MIRD dose estimate report for 2-[^18^F]FDG adopted a biokinetic compartmental model from Hays and Segall [4]. To set up this model, ^18^F activity concentrations in whole blood and plasma samples were measured in a gamma well counter and the retention data for lungs, liver and heart were derived by the quantification of activity from PET images. The activity in erythrocytes was inferred from the measurements in blood and plasma. For the distribution of 2-[^18^F]FDG in brain, a model previously published by Huang et al. [5] was incorporated in the biokinetic model of 2-[^18^F]FDG by Hays and Segall [4]. In addition, the time-integrated activity coefficients (TIACs) for kidneys, pancreas and spleen published by Mejia et al. [6] were employed in the MIRD dose estimate report [3] for dose calculation.

The model presented in ICRP Publication 128 is also based on the MIRD publication [3], together with additional data obtained by Deloar et al. [7] and by Mejia et al. [6]. As the vast majority of the models in [2], it is a descriptive model which identifies specific organs where the radiopharmaceutical is taken up and retained with characteristic half-times. The descriptive models neglect the initial phase of uptake and assume that certain fractions of the substance are taken up by organs and tissues instantaneously after the administration. So, for 2-[^18^F]FDG ICRP assumes immediate uptake of 8%, 4%, 3% and 5% of the activity in brain, heart wall, lungs and liver, respectively, at time-point zero with infinite retention. The latter indicates that there is no recycling of the substance and the 2-[^18^F]FDG activity in the respective organs decreases only due to the physical decay of ^18^F. The above-reported uptakes include for each organ 2-[^18^F]FDG activity in the organ parenchyma and in the blood within the organ. Eighty per cent of 2-[^18^F]FDG is distributed to other organs and tissues, of which thirty per cent is excreted into urine.

Currently, ICRP Task Group 36 “Radiation dose to patients in diagnostic nuclear medicine” (TG 36) is performing a revision of the reference models and dose coefficients for radiopharmaceuticals. The goal is to move from the current descriptive biokinetic models to more physiologically realistic compartmental models, like those already widely used for patient dosimetry in nuclear medicine and dosimetry from occupational and environmental radiation exposures in other ICRP reports [8–14]. Additionally, the absorbed dose coefficients in [2] were still computed using the mathematical phantoms developed by Cristy and Eckerman [15], which represented human anatomy in a simplified stylised way. The current ICRP reference computational models [16] provide a more realistic description of the anatomy and geometry of a human body. The formalism of the calculation of the effective dose coefficients also changed from that described in ICRP Publication 60 [17] to the one of ICRP Publication 103 [18]. Thus, an update of the dosimetric calculations according to the up-to-date methodologies is needed.

In the case of 2-[^18^F]FDG, quality checks conducted by the ICRP TG 36 and, independently, within a joint project of the European Radiation Dosimetry Group (EURADOS) and the European Association of Nuclear Medicine (EANM), revealed inconsistencies between the model published by MIRD [3, 4] and Hays and Segall [18] and the corresponding time-activity curves (TAC) and TIACs. Details of the EURADOS quality assurance study are described by Li et al. [19]. Additionally, the urinary excretion of 2-[^18^F]FDG experimentally measured by other authors [6, 20] was not in agreement with the predictions of the MIRD model.

These considerations indicated a need to revise the existing biokinetic models for 2-[^18^F]FDG and update the dosimetric calculations for this radiopharmaceutical to follow the current ICRP computational framework for internal dose assessment.

The objective of this study was thus to review available biokinetic data, collect own experimental data, when necessary, and propose an updated compartmental model for 2-[^18^F]FDG to be adopted for the revision of ICRP Publication 128.

## Material and methods

### Biokinetic data

The data used for the model definition are primarily measurements of ^18^F retention and excretion published by several authors [2, 4, 6, 7] after administration of 2-[^18^F]FDG. Note that strictly speaking the activity of ^18^F is measured. In the following, we assumed the activity of ^18^F to be the same as of 2-[^18^F]FDG.

Following data were considered for the model construction:

- *For blood:* time-resolved activity of 2-[^18^F]FDG recently obtained within the study by Brix et al. [21] from 32 healthy young adult volunteers and the data provided separately for plasma and erythrocytes of five adult volunteers by Hays and Segall [4];
- *For heart wall, liver, lungs:* activities of 2-[^18^F]FDG measured by Hays and Segall [4] up to 95 min post-injection (p.i.);
- *For brain, kidneys, spleen and pancreas:* data published by Mejia et al. [6] and Deloar et al. [7] for, respectively, 18 and six normal volunteers. The activity concentration in different organs reported by Mejia et al. [6] was scaled to the total activity using the organ masses of reference adult male [22].
- *For urine*: data published by Hays et al. [3], Mejia et al. [6] and Bach-Gansmo et al. [20] were used in the model fit. Due to substantial variations in these data, additional data were collected at Skåne University Hospital Malmö, Sweden and used here to validate the urinary excretion of 2-[^18^F]FDG predicted by the model.

The data used to set up the biokinetic model were corrected for physical decay of ^18^F.

#### Data collection protocol

For 23 patients (11 males, 12 females) referred to the Nuclear Medicine department for a 2-[^18^F]FDG PET/CT procedure, the activity concentration [kBq/ml] of ^18^F in the urinary bladder was determined from the whole-body PET images. The patients were informed in advance and written informed consent was obtained before imaging. The volume of the activity content [ml] in the urinary bladder was also achieved from the images. The ^18^F activity was estimated for each individual and normalised to the injected activity [%IA]. The PET scan was performed approximately 60 min post-injection, and each patient was encouraged to empty the urinary bladder just prior to imaging, as routine. The exact time of voiding and the subsequent imaging were recorded.

#### Data processing

The activity data obtained by quantification from PET images cannot distinguish between the ^18^F taken up by organ tissues and the ^18^F contained in blood contents of the organs. A very quick uptake and a fast decrease of ^18^F activity in lungs, liver, pancreas and spleen can be explained by the contribution of ^18^F activity in the blood within these organs. To consider this, we partitioned the measured activities between organ parenchyma and its blood content, calculated from the ICRP reference regional blood volume [22].

#### Model assumptions and calculations

The basic assumption of the proposed model is that transfer of material between model compartments can be described as a first-order-kinetics process, i.e. the flux (often denoted as “transfer rate”) of material transported from one compartment to another is proportional to the amount of material present in the originating compartment. The proportionality constants (transfer coefficients) are the unknown model parameters. Recycling is also considered, allowing for material to flow back and forth between compartments. Mathematically such model is described by a system of linear differential equations (see Additional file 1 for more details).

The model predictions were fit simultaneously to all available data using the Rosenbrock integrator option of SAAM II (The Epsilon Group, version 2.3) [23] to obtain the values of the unknown (adjustable) parameters. For the evaluation of the goodness of the fit, different statistical indicators are available. The SAAM II software provides among others:

- the uncertainties of the parameters, which correspond to one standard deviation; they are also expressed as relative uncertainty (coefficient of variation);
- the correlation coefficients, which give information on the interdependence of parameter pairs and give hint for possible simplifications of the model structure;
- the weighted residuals, i.e. the difference between experimental data and model prediction. Different variance models can be used to properly associate a weight to each datum. In the current analysis, the experimental uncertainties were used whenever available (data-based variance model); otherwise, the weight was defined using a model-based variance model with a relative uncertainty of 10% [23];
- the objective function, i.e. the function which is minimised by the software algorithm and depends on the weighted residuals;
- the values of the Akaike information criterion (AIC) and the Schwarz-Bayesian information criterion (BIC), which can be used to compare competing model structures and evaluate model order according to the principle of parsimony (the structure with lower AIC and BIC values is the most parsimonious one).

From the information provided it is furthermore possible to calculate the sum of the squared weighted residuals, which is distributed according to a chi-squared distribution with *N*–*M* degrees of freedom (*N* = number of data and *M* = number of free parameters). The goodness of fit can then be demonstrated comparing the calculated value of the sum of the squared weighted residuals with the theoretical chi-squared (*χ*^2^) distribution for the specific number of degrees of freedom. If the theoretical probability of observing a larger *χ*^2^ value than the estimated sum of the squared weighted residuals is less than 5% (i.e. if *p* < 0.05), the fit is considered rejected.

For quality assurance purposes, the developed biokinetic model with the fixed estimated transfer coefficients was implemented in a modified software version of IDAC-Iodide [24]. These independent calculations were used to validate the derived time-integrated activity coefficients (TIACs).

### Dosimetry

The number of disintegrations, or TIACs, was calculated for all model compartments by integration of the respective activity curve calculated assuming an intake of 1 MBq of 2-[^18^F]FDG under consideration of the physical decay of ^18^F. For each source region *S* the per cent difference ${\text{diff}}_{{{\text{TIAC}}}}^{S}$ of the revised TIAC relative to the corresponding TIAC of ICRP Publication 128 [2] was also computed as follows:

1 $${\text{diff}}_{{{\text{TIAC}}}}^{S} = \frac{{\left( {{\text{TIAC}}_{{{\text{revised}}}}^{S} - {\text{TIAC}}_{{{\text{ICRP128}}}}^{S} } \right) \times 100\% }}{{{\text{TIAC}}_{{{\text{ICRP128}}}}^{S} }}$$

The TIACs were then associated with the corresponding source regions of the reference voxel phantoms [16], and the absorbed organ dose coefficients in mGy/MBq were determined for reference adults using the programme IDAC-Dose 2.1 [25]. The effective dose was estimated according to the formalism of ICRP Publication 103 [18], including the updated tissue weighting factors presented there. The resulting dose coefficients for each target region of adult male were compared to the ones previously reported in ICRP Publication 128 [2] for adults. Analogously to the TIACs, the computed differences in dose coefficients were expressed in per cent of the corresponding values of ICRP Publication 128.

### Urinary excretion and dynamic bladder model

A dynamic bladder model was employed to estimate the absorbed dose to urinary bladder wall due to the decay of the activity contained in the urinary bladder [26]. This model considers changes in the urinary bladder volume due to the dynamic filling of the bladder and its emptying. A reference urinary production rate of 1600 ml/day and 1200 ml/day was assumed for male and female adults, respectively [22]. The volume of voided urine was set to 250 ml for males and to 200 ml for females, along with a residual urine volume of 10 ml. Thus, the voidings befall as soon as urinary bladder of adult male reached the volume of 260 ml, and 210 ml for adult female. This corresponds to the voiding intervals of 3.75 and 4 h for adult male and female, respectively, consistent with the assumption of 3.5 h voiding intervals for adults made in ICRP Publication 128. Since patients undergoing a PET scan with 2-[^18^F]FDG are usually encouraged to empty their bladder shortly before the image acquisition, additionally to the regular voiding intervals, the first voiding was set to occur at 45 min p.i. The initial urine volume of 100 ml at the time of the 2-[^18^F]FDG administration was assumed (this corresponds to a half-filled bladder). The SAFs for urinary bladder contents as source and urinary bladder wall as target region reported by Andersson et al. [27] were used. Andersson et al. approximated urinary bladder with spheres of various volumes, depending on the degree of filling the bladder with urine. A constant bladder wall mass has been assumed, whereby the volume of the cavity increases and the wall thickness decreases as bladder fills up. Thus, the SAFs computed by Andersson et al. [27] with Monte Carlo methods for monoenergetic photon and beta-particles of various energies account not only for a range of different bladder volumes, but also for the corresponding changes in the thickness of the urinary bladder wall. The contributions to the total absorbed dose coefficient for urinary bladder wall from the source regions other than urinary bladder contents were calculated using the SAFs simulated for the static bladder filled with 200 g of urine, as defined in the reference adult phantoms [16].

## Results

### Revised biokinetic model and model predictions

**Fig. 1:**
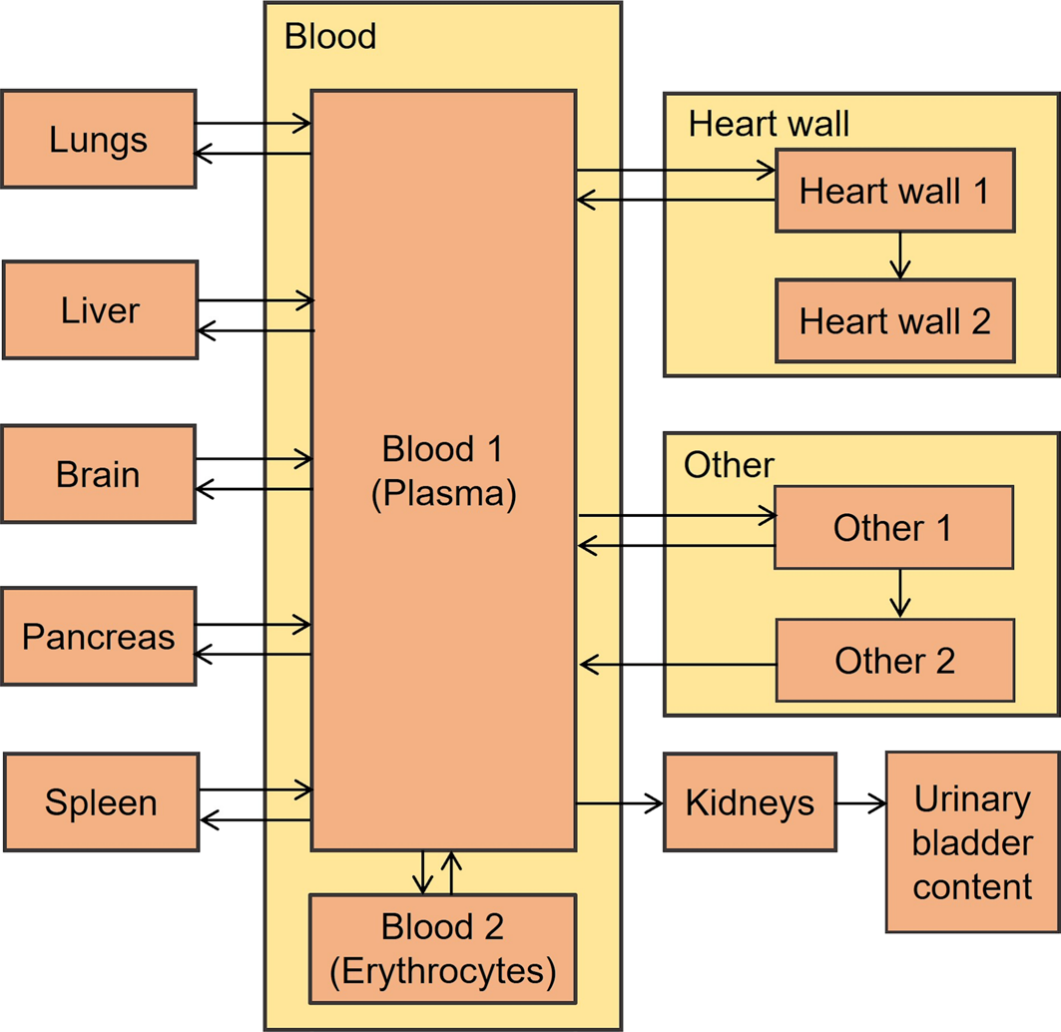
Structure of the proposed biokinetic model for 2-[^18^F]FDG

Based on the available data on 2-[^18^F]FDG uptake and distribution, following organs and tissues were considered as explicit source regions in the revised biokinetic model: blood, heart wall, brain, liver, lungs, pancreas, spleen, kidneys in addition to urinary bladder content. A preliminary version of the revised model was presented in [28] (abstract OP-0362). The final structure of the proposed biokinetic model is shown in Fig. 1. Blood is explicitly included as the central exchange compartment in the model. Analogously to Hays and Segall [4], blood is modelled by two sub-compartments—(1) plasma that transports the injected radiopharmaceutical to other body organs and tissues and (2) erythrocytes. To account for 2-[^18^F]FDG transported by plasma to body tissues besides these explicitly modelled source regions, an additional source region “Other” is considered (Fig. 1). Similar as in previous biokinetic models for 2-[^18^F]FDG [2, 4], the source region “Other” is modelled by two sub-compartments defining a short- and a long-term retention of 2-[^18^F]FDG. The experimental data [4] indicate a long-term retention of 2-[^18^F]FDG in heart wall. Thus, two sub-compartments are also used for heart wall to describe this observed behaviour. Best fit of the model to the experimental data was obtained when the transfer coefficient from “Heart wall 2” back to blood reached zero, meaning that the retention in “Heart wall 2” is very long compared to the half-life of ^18^F. Hence, this pathway was removed from the model structure. The urinary excretion of 2-[^18^F]FDG is described by a transfer from plasma to kidneys compartment and a subsequent flux from kidneys to urinary bladder content.

**Fig. 2:**
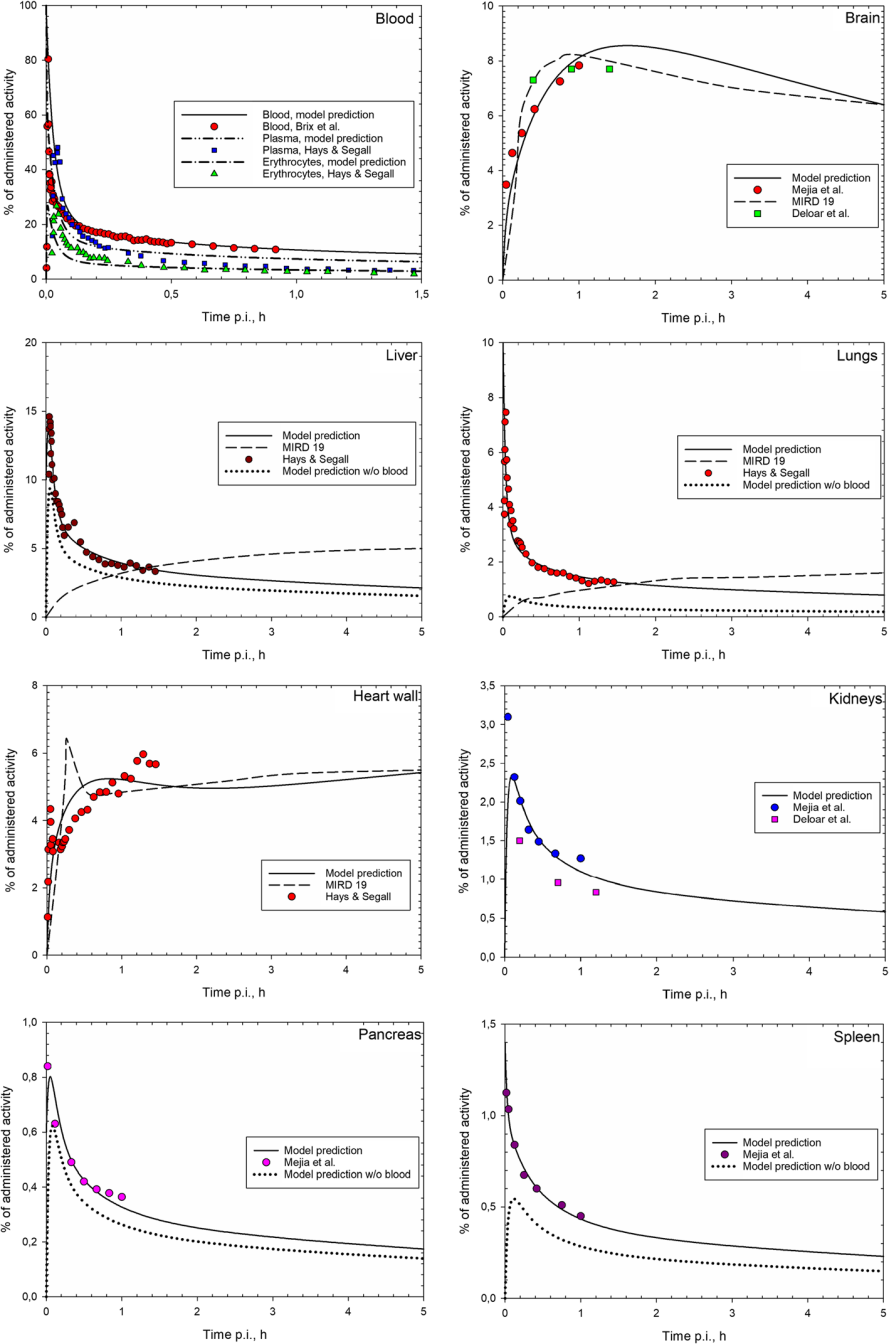
Decay-corrected TACs in source regions predicted by the proposed biokinetic model along with the experimental data (scattered points; decay-corrected to the time of injection). For comparison, TACs reported in MIRD dose estimate report 19 [3] are given in dashed lines. For liver, lungs, pancreas and spleen, the TACs predicted by the proposed model for organ parenchyma only (i.e. without organ blood content) are shown in dotted lines. Activity of 2-[^18^F]FDG is given in % of administered activity

The structure presented is the one that satisfied the principle of parsimony, having the lowest values of the information criterion and of the objective function. The transfer coefficients estimated for the model structure of Fig. 1 along with their standard deviations and the coefficients of variation are listed in Table 1. The uncertainties of the transfer coefficients resulted from the model fit under consideration of the uncertainties of time-activity data as described in the section “Material and Methods”. The corresponding predictions of the revised biokinetic model along with the experimental data are displayed in Fig. 2. The sum of the squared weighted residuals amounts to 212, the corresponding *p* value for 194 degree of freedom—to 0.18, i.e. the fit is considered adequate. As explained in the section “Material and Methods”, the curves of Fig. 2 show the biokinetics only, without the physical decay of ^18^F, and are denoted as “decay-corrected” time-activity curves (TACs). The proposed method to consider the contribution of 2-[^18^F]FDG activity in blood within organs allows modelling of TACs separately for organ with blood and for organ parenchyma. The latter predictions are shown in Fig. 2 as dotted lines for liver, lungs, pancreas and spleen. For highly vascularised organs as, e.g. lungs, the total measured activity obtained from PET scans almost solely consists of the 2-[^18^F]FDG activity in blood within lungs (Fig. 2).

**Table 1:** Transfer coefficients [h^−1^] for the revised biokinetic model for 2-[^18^F]FDG

From compartment	To compartment	Transfer coefficient [h^−1^] (estimated value ± standard deviation)	Coefficient of variation, %
Blood 1 (Plasma)	Blood 2 (Erythrocytes)	201 ± 20	10.0
Blood 1	Brain	1.12 ± 0.11	9.8
Blood 1	Lungs	0.43 ± 0.33	76.7
Blood 1	Liver	8.08 ± 0.98	12.1
Blood 1	Heart wall 1	1.19 ± 0.21	17.6
Blood 1	Other 1	25.4 ± 0.4	1.6
Blood 1	Kidneys	1.32 ± 0.23	17.4
Blood 1	Pancreas	0.39 ± 0.08	20.5
Blood 1	Spleen	0.28 ± 0.03	10.7
Blood 2	Blood 1	441 ± 26	5.9
Brain	Blood 1	0.80 ± 0.14	17.5
Heart wall 1	Blood 1	2.06 ± 0.77	37.4
Heart wall 1	Heart wall 2	0.20 ± 0.12	60.0
Kidneys	UB contents	9.20 ± 1.74	18.9
Liver	Blood 1	21.0 ± 3.7	17.6
Lungs	Blood 1	9.6 ± 7.7	80.2
Other 1	Blood 1	5.30 ± 0.91	17.2
Other 1	Other 2	0.84 ± 0.52	61.9
Other 2	Blood 1	0.78 ± 0.54	69.2
Pancreas	Blood 1	11.2 ± 2.6	23.2
Spleen	Blood 1	7.73 ± 1.07	13.8

*UB* urinary bladder

**Fig. 3:**
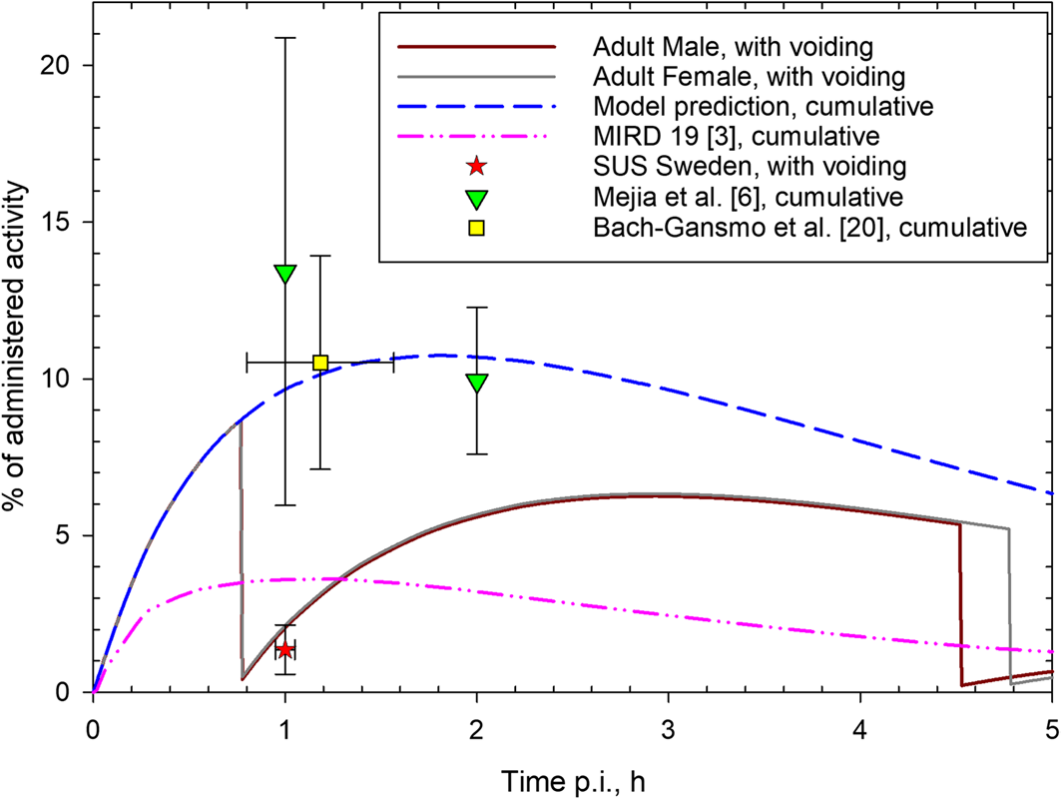
TACs in source region urinary bladder contents. The blue dashed line gives the TAC under the assumption of no voiding, i.e. it corresponds to the cumulative excretion of 2-[^18^F]FDG in urine. The red and grey solid lines display the TACs as predicted by the dynamic bladder model with the first voiding at 45 min p.i. for male and female adults, respectively. The purple dashed-dotted line gives the prediction of the MIRD model [3] (cumulative excretion). The red star corresponds to the average value of the bladder activities assessed in this study (SUS: Skåne University Hospital); the green triangles and the yellow square—to the cumulative excretion reported by Mejia et al. [6] and Bach-Gansmo et al. [20], respectively. All values are expressed as % of administered activity, and not corrected for physical decay

The urinary bladder data assessed in this study are reported in Table 2 and in Additional file 2: Table S2. According to the experimental protocols, the first voiding occurred at 44 ± 3 min (range 40–49), with the subsequent PET imaging taken approximately 1 h after 2-[^18^F]FDG administration. The average value of the measurements is shown in Fig. 3, together with the prediction of the dynamic bladder model and other data from the literature. The cumulative urinary excretion by Mejia et al. is assumed to be described by the TAC for urinary bladder when emptying is neglected.

**Table 2:** Collected urinary bladder data

Patient #	M/F	% IA^a^	First voiding	
			Min p.i	Min prior to start of whole-body scan^b^
1	M	0.6	43	19
2	M	1.4	46	10
3	F	1.1	42	18
4	F	2.0	41	22
5	M	2.0	42	25
6	F	1.5	40	18
7	F	2.5	40	22
8	M	1.2	46	13
9	M	2.3	48	14
10	M	1.9	42	14
11	M	0.8	48	8
12	F	0.9	49	11
13	F	0.9	44	19
14	M	1.2	44	14
15	F	1.1	43	18
16	M	3.6	41	21
17	F	3.1	43	15
18	F	1.5	46	9
19	F	1.3	44	17
20	F	1.0	44	14
21	F	1.1	41	17
22	M	0.6	44	12
23	M	0.5	48	11

^a^Not decay-corrected

^b^Urinary bladder was imaged 1.5 min after the start of whole-body scan

Based on the estimated TACs, the TIACs for all source regions were calculated (Table 3). The independent quality control calculations of TIACs resulted in identical values for all source regions except “Kidneys” and “Other”. For the latter source regions, the corresponding relative per cent differences amounted to 0.38% and 0.78%, respectively. Table 3 also shows the difference between the revised TIACs and those given in ICRP Publication 128. No differences in TIACs can be calculated for the source regions blood, kidneys, pancreas and spleen, since they were not included in the model of ICRP Publication 128. TIACs for the source region “Other” cannot be compared, since the corresponding source regions comprise different organs and tissues.

**Table 3:** TIACs [MBq-h/MBq] in the source regions for 2-[^18^F]FDG

Source region	TIAC, MBq-h/MBq	Difference: revised model versus ICRP 128
Blood	2.78E−01	N/A
Brain	1.82E−01	− 13%
Lung tissue	8.25E−03	− 90%
Liver	7.23E−02	− 44%
Heart wall	1.32E−01	+ 20%
Kidneys	2.63E−02	N/A
Pancreas	6.44E−03	N/A
Spleen	6.60E−03	N/A
UB contents^a^	3.06E−01	+ 18%
UB contents^b^	3.27E−01	+ 26%
Other	1.28E + 00	− 25%

^a^For adult male

^b^For adult female

### Dosimetry

Table 4 shows the calculated dose coefficients for absorbed doses to all target organs and tissues and for the effective dose.

**Table 4:** Dose coefficients for absorbed organ doses [mGy/MBq] and the effective dose [mSv/MBq] for adults administered with 2-[^18^F]FDG

Organ	Adult male (revised model) [mGy/MBq]	Adult female (revised model) [mGy/MBq]	Adult ICRP 128 [mGy/MBq]	Difference: adult male (revised model) versus ICRP 128
Adrenals	1.4E−02	1.6E−02	1.2E−02	+ 13%
Brain	3.0E−02	3.3E−02	3.8E−02	− 21%
Breast	7.6E−03	9.7E−03	8.8E−03	− 13%
Colon wall	1.2E−02	1.5E−02	1.2E−02	+ 1%
Endosteum (bone surface)	1.0E−02	1.2E−02	1.1E−02	− 7%
Extrathoracic region	7.6E−03	8.5E−03	N/A	N/A
Gallbladder wall	1.1E−02	1.3E−02	1.3E−02	− 14%
Heart wall	6.5E−02	8.4E−02	6.7E−02	− 3%
Kidneys	2.0E−02	2.3E−02	1.7E−02	+ 16%
Liver	1.5E−02	1.8E−02	2.1E−02	− 29%
Lungs	1.3E−02	1.7E−02	2.0E−02	− 37%
Lymphatic nodes	1.3E−02	1.4E−02	N/A	N/A
Muscle	8.3E−03	1.0E−02	1.0E−02	− 17%
Oesophagus	1.5E−02	1.7E−02	1.2E−02	+ 23%
Oral mucosa	8.8E−03	9.9E−03	N/A	N/A
Ovaries^a^	N/A	2.4E−02	1.4E−02	+ 71%
Pancreas	1.6E−02	1.8E−02	1.3E−02	+ 23%
Prostate	2.7E−02	N/A	N/A	N/A
Red (active) bone marrow	1.5E−02	1.8E−02	1.1E−02	+ 33%
Salivary glands	7.8E−03	9.7E−03	N/A	N/A
Skin	6.3E−03	7.7E−03	7.8E−03	− 19%
Small intestine wall	1.3E−02	1.7E−02	1.2E−02	+ 8%
Spleen	1.4E−02	1.7E−02	1.1E−02	+ 31%
Stomach wall	1.2E−02	1.3E−02	1.1E−02	+ 10%
Testes	8.6E−03	N/A	1.1E−02	− 22%
Thymus	9.8E−03	1.2E−02	1.2E−02	− 18%
Thyroid	9.1E−03	1.1E−02	1.0E−02	− 9%
Urinary bladder wall	7.5E−02	9.2E−02	1.3E−01	− 43%
Uterus/cervix^a^	N/A	3.3E−02	1.8E−02	+ 82%
Effective dose ICRP 60 (mSv/MBq)			1.9E−02	
Effective dose ICRP 103 (mSv/MBq)	1.7E−02			

^a^For these organs, the differences are expressed to Adult Female

For comparison, the dose coefficients for absorbed organ doses and the effective dose from ICRP Publication 128 along with the differences to the revised Adult Male dose coefficients computed in this work are also listed in Table 4. Note that the effective dose coefficient of ICRP Publication 128 was computed according to the formalism and the tissue weighting factors of ICRP Publication 60 [17], which differ from the ones of ICRP Publication 103 [18] used in this revision. Therefore, no percentage difference of the effective dose coefficients is given in the table. The organs receiving the highest absorbed dose were urinary bladder wall, heart wall and brain with absorbed dose coefficients of 7.5E−02, 6.5E−02 and 3.0E−02 mGy/MBq, respectively. In all three cases, the revised doses were lower than the ones currently given in ICRP Publication 128, the differences being − 43%, − 3% and − 21%, respectively. The highest increase in absorbed dose coefficients was observed for uterus (+ 82%) and ovaries (+ 71%). The revised effective dose coefficient amounted to 1.7E−02 mSv/MBq.

## Discussion

The revised compartmental model presented in this work describes the distribution of 2-[^18^F]FDG in a more physiologically realistic way compared to the descriptive biokinetic model previously reported in ICRP Publication 128 and includes recent measurements of activity in blood and urinary bladder. Important features in the revised model are the presence of blood as a central compartment that, after an intravenous injection, transfers 2-[^18^F]FDG to other body organs and tissues, and the explicit inclusion of pancreas and spleen as source regions, which were not considered in ICRP Publication 128. The source regions in the revised model were selected based on the available biodistribution data for 2-[^18^F]FDG. New experimental data obtained with, e.g. whole-body PET might help to evaluate whether the selection of source regions was adequate enough or further regions could be modelled as distinct sources. No indication of organs and tissues, besides those considered in this study, showing an uptake of 2-[^18^F]FDG notably higher than a general level of ^18^F activity in the body, was found in the currently published works though. The derived very quick transfer from plasma to erythrocytes and back shows that, instead of plasma, the whole blood can be considered as central exchange compartment in biokinetic studies of 2-[^18^F]FDG, as also mentioned by other authors [4]. Note that the incorporation of second sub-compartments for source regions “Heart wall” and “Other” was justified by the presence of a long-term retention of 2-[^18^F]FDG in these regions and a need to obtain accurate TACs and does not have an obvious physiological rationale.

The proposed biokinetic model showed a good description of the experimental data. The highest difference in TIACs compared to ICRP Publication 128 was observed for lungs and liver (− 90% and − 44%, respectively). It should be considered that lungs and liver are highly vascularised organs. In the revised model presented here, the contribution of 2-[^18^F]FDG activity in the blood within these organs was modelled separately from the 2-[^18^F]FDG activity in the parenchyma. The reported values represent thus TIACs in the parenchyma only, without the contribution due to the activity in the regional blood volumes of these organs (the same holds here for pancreas and spleen). On the contrary, in ICRP Publication 128 blood was not considered as an explicit source region and the provided TIACs include 2-[^18^F]FDG activity in both organ parenchyma and blood. This explains the observed differences in the TIACs for lungs and liver. (Pancreas and spleen were not present in the previous model structure.) The separate modelling of the contribution of blood to the activity measured in a given organ enabled to estimate more realistically the TIACs in the organ parenchyma. Moreover, considering blood as a distinct source region overcomes a possible underestimation of organ-absorbed doses for non-source organs with substantial mass fraction of blood content [29].

The radiopharmaceutical 2-[^18^F]FDG is to a large extent eliminated via the urine. The assumption of voiding intervals affects the predicted TIAC in the urinary bladder content. Another improvement of the revised model was thus the usage of the dynamic bladder model that allows more realistic simulations of filling and emptying the urinary bladder and, thus, of the urinary excretion of 2-[^18^F]FDG and the corresponding absorbed dose to urinary bladder wall. Generally, any arbitrary voiding interval or fixed voiding scheme can be assumed to calculate the TIACs in the urinary bladder content. The ICRP Publication 128 assumes a constant voiding interval for adults of 3.5 h. However, for 2-[^18^F]FDG scans the patients are often hydrated and the first voiding occurs just before the image acquisition (45–60 min after administration). Therefore, the first voiding at 45 min was assumed in the calculations of this study. In spite of the early first voiding, the TIACs with the revised model are about 20–25% higher than the one calculated in ICRP Publication 128. This can be explained by the fact that the MIRD model (the basis of those calculations) notably underestimated the excretion data (Fig. 3). Urinary bladder activity measured at 2 h p.i. by Jones et al. [30] is consistent with the data by Mejia et al. [6] and well described by the model of this study. The cumulative bladder excretion reported in [31] lies between the MIRD and the revised models and amounts to 5.8–7.1%IA at 1 h p.i. (compared to ca. 3.6 and 9.7%IA predicted by the MIRD and the revised models, respectively). The urinary excretion of 2-[^18^F]FDG as predicted by the model showed a good agreement with the ^18^F activity in urinary bladder as obtained in the SUS studies (SUS data, Fig. 3); while the volumes of urinary bladder contents at 1 h p.i. (SUS data, Additional file 2: Table S2) were for most patients notably higher than those predicted by the dynamic bladder model under the assumption of a reference urinary production rate [22] and a residual urine volume of 10 ml. This could be due to (1) only partial emptying the bladder by the patients at the first voiding and, consequently, larger residual urine volumes and (2) higher urinary production rate because of hydration before the 2-[^18^F]FDG diagnostic scan. Underestimation of urine volumes can lead to an overestimation of the absorbed dose to bladder wall, since SAFs for bladder content as source and bladder wall as target regions decrease with increasing the volume of bladder content. Thus, the reported absorbed dose coefficient for bladder wall is possibly a conservative estimate.

The organ dose coefficients were calculated according to the latest dosimetric framework of the ICRP, in particular making use of the reference anthropomorphic voxel phantoms instead of the stylized mathematical phantoms employed in ICRP Publication 128. The revised absorbed dose coefficients for uterus and ovaries were notably higher compared to those reported in ICRP Publication 128 partly due to the higher revised TIAC for urinary bladder contents and also because of different (generally more realistic) anatomical description of the geometry in the voxel phantoms used in the calculations of the cross-fire energy deposition.

The dose to the urinary bladder wall is notably lower (− 47%) than in the previous calculations although the TIAC in the bladder content is higher. In this case, no direct comparison can be made due to several reasons. First, the dynamic bladder model employed here was used in a combination with SAFs that vary with varying bladder volume (and thickness of the bladder wall). In addition, the new dosimetric approach is based on a realistic assessment of the energy deposition in the organ wall also for beta-particles, and not on the previously used simplified assumption that half of the energy of beta-particles is absorbed in the wall. To evaluate the impact of these features, some additional calculations were made. First of all, the contributions to the absorbed dose to urinary bladder wall from the source region urinary bladder contents were computed also for the static bladder model, which assumes fixed volume of the bladder and thickness of the bladder wall. Using the bladder TIAC from Table 3, values of 5.7E−02 mGy/MBq and 4.3E−02 mGy/MBq were obtained for photons and 9.6E−03 mGy/MBq and 9.1E−03 mGy/MBq for beta-particles for the dynamic and the static bladder models, respectively. The use of smaller bladder volumes for emptied or not completely filled bladder in the dynamic model would thus lead to a higher dose to the bladder. However, the more realistic treatment of the beta-particles energy deposition in the bladder wall causes a drastic reduction of the beta contribution to the bladder dose of nearly an order of magnitude. (It was equal to 8.9E−02 mGy/MBq according to the previous assumptions.) The present calculations with more realistic biokinetic and dosimetric models thus show that, from one hand, the static bladder model underestimated the dose to bladder wall (especially from the photon component) and, from the other hand, that the previous simplistic assumptions on beta-particles dosimetry led to an unjustified overestimation of the absorbed dose to the bladder wall in spite of the fact that the excretion curve of 2-[^18^F]FDG was underestimated by the MIRD model, as shown in Fig. 3.

Finally, the influence of the selected initial settings for the dynamic bladder model on the absorbed dose to the urinary bladder wall was also analysed. Firstly, assuming an initial bladder volume of 10 ml (this corresponds to having emptied the bladder just before the time of 2-[^18^F]FDG injection) instead of a half-full bladder, as in our reference calculations, leads to an increase of the urinary bladder wall dose by approximately 10% despite the same TIAC value. This increase is due to the slightly larger SAFs for smaller bladder volumes. Secondly, if no forced voiding at 45 min p.i. is assumed and the voiding pattern befalls according to the dynamic bladder model (every 3,75 h for adult male and every 4 h for adult female), the urinary bladder wall dose would increase by 25%, due to the higher TIAC values. This shows the positive effect on dose (and not only on imaging) of forcing a bladder voiding before the first scan. Similarly, hydration helps to decrease the absorbed dose to the bladder wall by diluting the activity in a larger urine volume and inducing a higher voiding frequency. The dose reduction is, however, not substantial and amounts to about 5% in the case considered here.

Recently, Hu et al. [31] investigated an impact of TACs measured with a total-body high-sensitive PET/CT scanner up to 8 h p.i. on dosimetry of 2-[^18^F]FDG. The TIAC for brain in [31] was notably higher compared to the ones reported here and by other authors [2, 4, 6, 7, 32]. Brain-absorbed dose does not notably influence the effective dose though, since brain has a tissue weighting factor of only 0.01 [18]. Hu et al. obtained the effective dose coefficient of 1.4E−02 mSv/MBq compared to 1.7E−02 mSv/MBq in this work.

The calculation of the effective dose coefficients according to the current recommendations of the ICRP [18] is affected in a complicated and partially opposite way by the simultaneous combination of the different methodological improvements implemented in this work: revised and physiologically more realistic biokinetic model, improved and realistic description of the urinary excretion, anatomically realistic reference voxel phantoms, improved assumptions for the calculations of the energy deposition in the target organs, especially for beta-particles, revised values of radiation and tissue weighting factors. As a result, the revised effective dose coefficient is about 10% lower than the one in ICRP Publication 128 (1.7E−02 vs 1.9E−02 mSv/MBq).

## Conclusion

This study proposes a revised compartmental model for 2-[^18^F]FDG based on the biokinetic data reported by different authors and the collected urinary excretion data. The dosimetric calculations were updated to follow the current ICRP computational framework for internal dose assessment. The developed compartmental model and the provided dose coefficients will be used in the revision of ICRP Publication 128 and are expected to improve reference dosimetry of patients administered with 2-[^18^F]FDG.

## Supplementary Information

**Additional file 1**. Mathematical background for the compartmental structure used to describe the biokinetics of 2-[^18^F]FDG.

**Additional file 2**. Urinary bladder data collected in the current study.

## Data Availability

The data generated within the current study are available from the corresponding author on reasonable request.
