# Supplementary material for: A revised compartmental model for biokinetics and dosimetry of 2-[18F]FDG
Source: EJNMMI Phys. 2023 Feb 8;10:10. doi: 10.1186/s40658-023-00528-9 (PMC9908780; doi:10.1186/s40658-023-00528-9)
Supplement: Supplementary file 1 — Additional file 1. Mathematical background for the compartmental structure used to describe the biokinetics of 2-[18F]FDG. [file 40658_2023_528_MOESM1_ESM.docx]

**Additional file 1.**

**Mathematical background for the compartmental structure used to describe the biokinetics of 2-[^18^F]FDG**

First-order kinetics is assumed in the proposed compartmental model, meaning that the flux (often denoted as “transfer rate”) of material transported from one compartment to another compartment is proportional to the amount of material present in the originating compartment:

| $F_{i}^{\text{in}}\left( t \right)=\sum_{\begin{aligned} j=1 \\ j\neq i \end{aligned}}^{N} k_{ij}A_{j}\left( t \right)$ | (1) |
| --- | --- |
| $F_{i}^{\text{out}}\left( t \right)=\sum_{\begin{aligned} j=0 \\ j\neq i \end{aligned}}^{N} k_{ji}A_{i}\left( t \right)$ | (2) |

where $F_{i}^{\text{in}}$ and $F_{i}^{\text{out}}$ are the transfer rates at which material enters and leaves the compartment *i*, respectively; *N* is the number of compartments in the model; $A_{j}\left( t \right)$ and $A_{i}\left( t \right)$ in this study denote the activities in compartments *j* and *i*, respectively. The proportionality constants (transfer coefficients) *k_ij_* and *k_ji_* are the unknown model parameters. For example, the parameter *k_ij_* is the transfer coefficient for material transport from compartment *j* to compartment *i*. In equation (2) the case *j*=0 describes the loss of material from compartment *i* to the external environment, e.g. an excretion from human body. Recycling is also considered, allowing for material to flow back and forth between compartments. Mathematically such model is described by a system of linear differential equations:

| $\frac{dA_{i}(t)}{dt}=u_{i0}+\sum_{\begin{aligned} j=1 \\ j\neq i \end{aligned}}^{N} k_{ij}A_{j}\left( t \right)-\sum_{\begin{aligned} j=0 \\ j\neq i \end{aligned}}^{N} k_{ji}A_{i}\left( t \right)$ | (3) |
| --- | --- |

for each $i=\overline{1,N}$. The variable $u_{i0}$ represents an input of material from external environment to compartment *i*, e.g. in this study, an administration of a radiopharmaceutical.

Table S1. Symbols used in the system of differential equations for the developed compartmental biokinetic model for 2-[^18^F]FDG (see Figure 1 of the main text).
*Activity coefficient is an activity given as fraction of injected activity.
**UB is urinary bladder.
^⸸^Normalized to injected activity, it is always equal to 1.
^‡^It is equal to 0.379 [h^-1^]

| Activity coefficient* in respective compartment  of the biokinetic model | Symbol |
| --- | --- |
| Blood 1 (Plasma) | $x_{1}$ |
| Blood 2 (Erythrocytes) | $x_{12}$ |
| Brain | $x_{2}$ |
| Heart wall 1 | $x_{3}$ |
| Heart wall 2 | $x_{32}$ |
| Kidneys | $x_{4}$ |
| Liver | $x_{5}$ |
| Lungs | $x_{6}$ |
| Other 1 | $x_{7}$ |
| Other 2 | $x_{72}$ |
| Pancreas | $x_{8}$ |
| Spleen | $x_{9}$ |
| UB** contents | $x_{10}$ |
| Known injected activity coefficient^⸸^ | $u_{1}$ |
| Decay constant of ^18^F^‡^ | $\lambda$ |

Assuming the nomenclature of Table S1, the system of ordinary differential equations used to mathematically describe the kinetics of 2-[^18^F]FDG is given below:

$$\left\{ \begin{aligned} \begin{matrix} \frac{dx_{1}}{dt}=u_{1}-\left( k_{2,1}+k_{3,1}+k_{4,1}+k_{5,1}+k_{6,1}+k_{7,1}+k_{8,1}+k_{9,1}+k_{12,1} \right)x_{1}+k_{1,2}x_{2}+k_{1,3}x_{3}+ \\ +k_{1,5}x_{5}+k_{1,6}x_{6}+k_{1,7}x_{7}+k_{1,72}x_{72}+k_{1,8}x_{8}+k_{1,9}x_{9}+k_{1,12}x_{12}-\lambda x_{1}; \\ \begin{matrix} \frac{dx_{2}}{dt}=k_{2,1}x_{1}-k_{1,2}x_{2}-\lambda x_{2}; \\ \frac{dx_{3}}{dt}=k_{3,1}x_{1}-k_{1,3}x_{3}-k_{32,3}x_{3}-\lambda x_{3}; \\ \frac{dx_{4}}{dt}=k_{4,1}x_{1}-k_{10,4}x_{4}-\lambda x_{4}; \end{matrix} \end{matrix} \\ \begin{matrix} \frac{dx_{5}}{dt}=k_{5,1}x_{1}-k_{1,5}x_{5}-\lambda x_{5}; \\ \frac{dx_{6}}{dt}=k_{6,1}x_{1}-k_{1,6}x_{6}-\lambda x_{6}; \\ \begin{matrix} \frac{dx_{7}}{dt}=k_{7,1}x_{1}-k_{1,7}x_{7}-k_{72,7}x_{7}-\lambda x_{7}; \\ \frac{dx_{8}}{dt}=k_{8,1}x_{1}-k_{1,8}x_{8}-\lambda x_{8}; \\ \begin{matrix} \frac{dx_{9}}{dt}=k_{9,1}x_{1}-k_{1,9}x_{9}-\lambda x_{9}; \\ \frac{dx_{10}}{dt}=k_{10,4}x_{4}-\lambda x_{10}; \\ \begin{matrix} \frac{dx_{12}}{dt}=k_{12,1}x_{1}-k_{1,12}x_{12}-\lambda x_{12}; \\ \frac{dx_{72}}{dt}=k_{72,7}x_{7}-k_{1,72}x_{72}-\lambda x_{72}; \end{matrix} \end{matrix} \end{matrix} \end{matrix} \end{aligned} \right.$$
