# Supplementary material for: A revised compartmental model for biokinetics and dosimetry of 2-[18F]FDG
Source: EJNMMI Phys. 2023 Feb 8;10:10. doi: 10.1186/s40658-023-00528-9 (PMC9908780; doi:10.1186/s40658-023-00528-9)
Supplement: Supplementary file 2 — Additional file 2. Urinary bladder data collected in the current study. [file 40658_2023_528_MOESM2_ESM.docx]

**Additional file 2.**

**Urinary bladder data collected in the current study**

Table S2. Activity concentration and volume of urinary bladder contents measured in the SUS studies. The reported time corresponds to the start of whole-body PET scan for each patient, and the urinary bladder was imaged 1.5 minutes thereafter. The activity concentrations are not corrected for physical decay of ^18^F.

| Patient # | Time  of PET scan, [min p.i.] | Activity concentration in UB, [kBq/ml] | Volume, [cm^3^] | IA, [MBq] |
| --- | --- | --- | --- | --- |
|  |  |  |  |  |
| 1 | 62 | 13.7 | 130 | 319 |
| 2 | 56 | 154 | 36.5 | 407 |
| 3 | 60 | 13.8 | 233 | 302 |
| 4 | 63 | 15.5 | 363 | 279 |
| 5 | 67 | 100 | 63.4 | 322 |
| 6 | 58 | 19.4 | 314 | 407 |
| 7 | 62 | 39.1 | 201 | 319 |
| 8 | 59 | 32.1 | 111 | 303 |
| 9 | 62 | 28.8 | 304 | 375 |
| 10 | 56 | 55.6 | 123 | 366 |
| 11 | 56 | 52.5 | 37.5 | 232 |
| 12 | 60 | 29.4 | 97.8 | 316 |
| 13 | 63 | 77.6 | 43.4 | 378 |
| 14 | 58 | 16.3 | 192 | 262 |
| 15 | 61 | 13.3 | 155 | 193 |
| 16 | 62 | 65.7 | 181 | 332 |
| 17 | 58 | 58.2 | 157 | 291 |
| 18 | 55 | 31.7 | 122 | 259 |
| 19 | 61 | 21.0 | 150 | 237 |
| 20 | 58 | 34.2 | 85.2 | 302 |
| 21 | 58 | 75.0 | 29.6 | 199 |
| 22 | 56 | 16.4 | 127 | 358 |
| 23 | 59 | 83.4 | 22.7 | 348 |
